# Eight years of single-molecule localization microscopy

**DOI:** 10.1007/s00418-014-1184-3

**Published:** 2014-02-05

**Authors:** Teresa Klein, Sven Proppert, Markus Sauer

**Affiliations:** Department of Biotechnology and Biophysics, Biocenter, Julius Maximilian University Würzburg, Am Hubland, 97074 Würzburg, Germany

**Keywords:** Super-resolution imaging, Localization microscopy, *d*STORM, PALM, Live-cell imaging, 3D imaging

## Abstract

Super-resolution imaging by single-molecule localization (localization microscopy) provides the ability to unravel the structural organization of cells and the composition of biomolecular assemblies at a spatial resolution that is well below the diffraction limit approaching virtually molecular resolution. Constant improvements in fluorescent probes, efficient and specific labeling techniques as well as refined data analysis and interpretation strategies further improved localization microscopy. Today, it allows us to interrogate how the distribution and stoichiometry of interacting proteins in subcellular compartments and molecular machines accomplishes complex interconnected cellular processes. Thus, it exhibits potential to address fundamental questions of cell and developmental biology. Here, we briefly introduce the history, basic principles, and different localization microscopy methods with special focus on *direct* stochastic optical reconstruction microscopy (*d*STORM) and summarize key developments and examples of two- and three-dimensional localization microscopy of the last 8 years.

## Introduction

Due to the availability of efficient fluorescent probes and labeling recipes, fluorescence microscopy allows the direct observation of cellular processes in fixed and living cells as well as in complete organisms with molecular specificity and high temporal resolution in three dimensions (Giepmans et al. 2006; Haugland 2005; Lippincott-Schwartz and Patterson 2009; Pawley 2006). Due to the wave nature of light, however, the spatial resolution is limited to about half of the wavelength of the light in the imaging plane (Abbe 1873; Rayleigh 1903), that is, conventional confocal and wide-field fluorescence microscopy does not provide insight into the structural and dynamic organization of biological samples such as vital protein assemblies and machineries with a size of a few tens of nanometers.

Within recent years, different far-field microscopic approaches have been introduced that have found a way to bypass the so-called diffraction barrier exploiting concepts to distinguish fluorescence emission of fluorophores in an additional dimension, e.g., absorption, fluorescence emission, and fluorescence lifetime (Betzig 1995; Heilemann et al. 2002; van Oijen et al. 1998), and thus localize their positions individually. However, the number of distinguishable spectroscopic characteristics of fluorophores is too low to distinguish thousands of fluorophores per diffraction-limited area that compose a typical biological structure. On the other hand, fluorescence emission of fluorophores is distinguishable also in time allowing the temporal separated fluorescence detection of fluorophores. Thus, the key to super-resolution imaging is confinement of the number of simultaneously fluorescing fluorophores. This is achieved in a deterministic way by generating a light pattern that prevents the emission of fluorophores in a defined area, as used by stimulated emission depletion (STED) (Dyba and Hell 2002; Hell and Wichmann 1994; Willig et al. 2006), and structured illumination microscopy (SIM) (Gustafsson 2000, 2005; Heintzmann and Cremer 1999; Kner et al. 2009) or in a stochastic way by temporally modulating the emission of individual fluorescent molecules by photoactivation, photoconversion, or photoswitching, as used in photoactivated localization microscopy (PALM) (Betzig et al. 2006; Manley et al. 2008; Shroff et al. 2007), fluorescence photoactivation localization microscopy (FPALM) (Hess et al. 2006, 2007), stochastic optical reconstruction microscopy (STORM) (Bates et al. 2007; Huang et al. 2008b; Rust et al. 2006), *direct* STORM (*d*STORM), (Heilemann et al. 2008, 2009; van de Linde et al. 2011b), and related localization microscopy approaches (Baddeley et al. 2009; Bock et al. 2007; Fölling et al. 2008; Geisler et al. 2007; Gunkel et al. 2009; Lemmer et al. 2008; Steinhauer et al. 2008; Vogelsang et al. 2009) (Table 1). Historically, localization microscopy started with the efforts of Lidke et al. (2005) to use the fluorescence intermittencies of semiconductor quantum dots for single-molecule localization. The authors envisioned that a high-resolution image consisting of individually localized points may be reconstructed and termed it “Pointillism” (Table 1). However, because blinking characteristics of quantum dots can be best described by a power law (Kuno et al. 2000), densely labeled structures are difficult to resolve. Alternatively, transient binding of fluorescent probes to the target structure (Giannone et al. 2010; Sharonov and Hochstrasser 2006) or the conversion of fluorogenic probes by catalysts (Roeffaers et al. 2009) can be exploited to bypass the diffraction limit by the reconstruction of a super-resolved image.

**Table 1:** Single-molecule localization microscopy techniques

Technique	Basic principle	Experimental details	References
Pointillism	Fluorescence intermittencies of emitters such as triplet-state blinking can be used to temporally separate fluorescence emission, localize emitters, and reconstruct a structure of interest (e.g., the cytoskeleton) by essentially painting them in a “*pointillistic*” fashion	Blinking of randomly distributed quantum dots on a coverslip (Qd655); ~1.000 photons per on-event; off-state lifetime too short to demonstrate usability to resolve cellular structure	Lidke et al. (2005)
PALM	*Photoactivated localization microscopy* uses photoactivation or photoconversion of fluorescent proteins to generate a sparse subset of fluorescent molecules per frame	Photoactivatable and photoconvertible fluorescent proteins (e.g., PAGFP, mEos, and PAmCherry); no special buffer additions required; typically, a few hundred photons per on-event; (Betzig et al. 2006) demonstrated first correlative PALM and electron microscopy images	Betzig et al. (2006), Manley et al. (2008), Shroff et al. (2007)
STORM	*Stochastic optical reconstruction microscopy* uses pairs of fluorophores (e.g., Cy3–Cy5) to photoswitch the reporter (Cy5) in the presence of thiols between a bright on- and a reduced off-state with long lifetime (> 1 s); the activator (Cy3) is used for efficient reactivation of the reporter	Standard fluorophore dye pairs (Cy5/Cy3 and Alexa 647/Cy3); addition of 10–100 mM of thiols such as mercaptoethylamine (MEA); oxygen depletion; off-state lifetimes of seconds to minutes; ~3.000 photons per on-event of Cy5/Alexa 647	Bates et al. (2005), Bates et al. (2007), Huang et al. (2008b), Rust et al. (2006)
FPALM	*Fluorescence photoactivation localization microscopy* uses photoactivation or photoconversion of fluorescent proteins to generate a sparse subset of fluorescent molecules per frame	Photoactivatable fluorescent proteins (e.g., PAGFP); no special buffer additions required; typically, a few hundred photons per on-state	Hess et al. (2006, 2007)
PALMIRA	*PALM with independently running acquisition* uses an asynchronous acquisition protocol and a fast camera that allows matching the camera frame time to the average on time of fluorophores	Fluorescent protein (rsFastLime) and standard fluorophore (Cy5); embedding in poly (vinyl-alcohol) (PVA) to prolong triplet-state lifetime; ~200 photons per on-state of rsFastLime, ~700 photons per on-state of Cy5	Bock et al. (2007), Geisler et al. (2007)
*d*STORM	*Direct STORM* photoswitches standard fluorescent probes in the presence of thiols between a bright on- and a reduced off-state with long lifetime (>1 s)	Standard fluorophores (cyanine, rhodamine, and oxazine dyes, e.g., ATTO- and Alexa dyes); addition of 10–100 mM of thiols such as mercaptoethylamine (MEA); off-state lifetimes of seconds to minutes; 500–5.000 photons per on-event dependent on dye	Heilemann et al. (2005, 2008, 2009), van de Linde et al. (2011b)
SPDM	*Spectral precision distance microscopy/spectral position determination microscopy* uses “reversible photobleaching” of fluorophores and fluorescent proteins under high irradiation intensities	Standard fluorophores and fluorescent proteins (e.g., YFP) mounted in an antifade reagent; irradiation intensities of 10 kW cm^−2^–1 MW cm^−2^; >1.000 photons detected per on-event of YFP molecules	Baddeley et al. (2009), Gunkel et al. (2009), Lemmer et al. (2008)
GSDIM	*Ground state depletion microscopy followed by individual molecule return* uses triplet and other metastable off-states of fluorophores for temporal separation of fluorescence emission of individual fluorophores	Standard fluorophores (ATTO 532, Alexa 488, Texas Red) and fluorescent proteins (e.g., EGFP and EYFP); oxygen depletion or embedding in poly(vinyl-alcohol) (PVA) to prolong off-state lifetime to 10–100 ms; 800–2.600 photons per on-event	Fölling et al. (2008), Hell (2009)
Blink Microscopy	Engineering on- and off-states by controlling the photophysics of the fluorophores by electron transfer reactions	Standard fluorophores (Cy5, ATTO 655, ATTO 680, ATTO 700); oxygen removal and addition of 100 μM ascorbic acid to quench the triplet state and generate reduced fluorophores with off-state lifetimes >20 ms; 70–700 photons per on-event	Steinhauer et al. (2008), Vogelsang et al. (2009)

Most techniques work optionally with a second laser (405 nm) to increase the reactivation efficiency of the on-state (the on-switching probability). In many cases, the reactivation efficiency of the readout laser is high enough to ensure a constant number of fluorophores residing in the on-state at any time of the experiment. With the exception of SPDM, all other localization microscopy techniques apply excitation intensities of a few kW cm^−2^ (typically 1–3 kW cm^−2^) for fluorescence readout and off-switching or photobleaching, respectively. The recovery or reactivation intensities are two to three orders of magnitude smaller

All stochastic single-molecule localization microscopy techniques (Table 1) share the same concept that only a sparse subset of fluorophores is allowed to stay in its fluorescent on-state at any time of the experiment. This is achieved by stochastic activation of individual fluorophores, single-molecule detection using a wide-field fluorescence microscope equipped with a sensitive charge-coupled device (CCD) camera, and precise position determination (localization), i.e., fitting of ideal point-spread functions (PSFs) to the measured photon distributions. As long as the distance between individual fluorophores residing simultaneously in their on-state (i.e., per frame) is larger than the distance resolvable by the microscope (>λ/2 on the CCD camera), individual fluorophores can be clearly localized. Since the localization precision (i.e., the accuracy of position determination of a single fluorophore) depends mainly on the number of collected photons, *N*, and on the standard deviation, *σ*, of the PSF, single fluorophores can be localized with an accuracy approximated by *σ*/*N* ^1/2^ for negligible background (Mortensen et al. 2010; Thompson et al. 2002).

## Single-molecule localization microscopy with synthetic fluorophores

While all single-molecule localization microscopy methods share the same concept of temporal separation of fluorescence emission, they use different concepts to accomplish this experimental requirement (Table 1). To ensure that only a sparse subset of fluorophores resides in the on-state at any time of the experiment and that each fluorophore defining the structure of interest is detected as individual emitter and thus precisely localized, the generation of stable off-states with lifetimes exceeding seconds is of outstanding importance. While this task is comparatively easy to accomplish using photoactivatable fluorescent proteins, which are non-fluorescent at the beginning of the experiment, the use of standard organic fluorophores requires the transfer of the majority of bright fluorophores to a long-lived, stable off-state. This succeeds for most Alexa and ATTO dyes—belonging to the cyanine, rhodamine, and oxazine dye classes—upon irradiation of an aqueous solution in the presence of millimolar concentrations of reducing thiols. This mechanism forms the basis for the *d*STORM concept (Heilemann et al. 2008, 2009; van de Linde et al. 2011b). The dyes are cycled between the singlet ground and first excited singlet state until the dye enters the triplet state. The triplet state can then be quenched to repopulate the singlet ground state either by oxygen or by the thiolat (the actual reducing species) to form a semi-reduced fluorophore radical anion, the fully reduced leuco form of the fluorophore, or a thiol adduct (Dempsey et al. 2009; van de Linde et al. 2011a). Finally, the singlet ground state is recovered by the oxidation of the reduced off-state, e.g., with molecular oxygen. Because the off-state of most organic fluorophores absorbs at ∼400 nm, photoinduced recovery can be applied to control the number of fluorophores residing in the on-state (van de Linde et al. 2011a).

Since *d*STORM is easy to perform, i.e., it works with commercial available fluorescent standard probes in aqueous solvents and standard protocols for immunostaining (Heilemann et al. 2009; van de Linde et al. 2008, 2009, 2011b, 2012, 2013), it is currently the most applied single-molecule localization microscopy concept using organic fluorophores. The simple thiol-assisted photoswitching mechanism (millimolar concentrations of thiols such as β-mercaptoethylamine, cysteine, or glutathione have to be added to the imaging buffer) and the availability of efficient open-source software for two-dimensional (2D) and three-dimensional (3D) multicolor localization microscopy (e.g., rapi*d*STORM) (Wolter et al. 2010, 2012) facilitated the acceptance and broad applicability of *d*STORM for different super-resolution imaging studies (Dempsey et al. 2011; Endesfelder et al. 2010, 2011; Kaminski Schierle et al. 2011; Lampe et al. 2012; Mattila et al. 2013; Rossy et al. 2013; Sillibourne et al. 2011; van de Linde et al. 2011b, 2012; Williamson et al. 2011; Wilmes et al. 2012; Zessin et al. 2012).

At the end of the day, the feasibility and reliability of different localization microscopy techniques has to be judged by test samples with well-defined molecular structure and composition. Among the various natural biological test samples, the nuclear pore complex (NPC) with its well-defined molecular composition, subdiffraction dimension, and eightfold symmetry holds a special position (Adams and Wente 2013). Because the NPC is composed of a defined number of proteins and exhibits a central channel with a diameter of ~40 nm, it is suited for testing of the achievable resolution of different super-resolution imaging methods and counting the number of molecules in a biological structure. The combination of raw *d*STORM localization data and particle averaging techniques as first demonstrated by Löschberger et al. (2012) can also be used to reconstruct average protein positions in multiprotein complexes such as the NPC with nanometer resolution (Szymborska et al. 2013). It neglects, however, structural heterogeneity of the sample by averaging individual signals—the strength of all single-molecule methods—and does not enhance the optical resolution.

Whereas super-resolution imaging or localization of fluorophore-labeled target molecules does not rely on the number of fluorophores attached to the molecule per se, the labeling density determines the achievable resolution of complex biological structures. According to the information theory, the required density of fluorescent probes must be high enough to satisfy the Nyquist–Shannon sampling theorem (Shannon 1949). In essence, this theorem states that the average distance between neighboring fluorophores (the sampling interval) must be at least two times smaller than the desired spatial resolution. For example, to resolve structural features with a spatial resolution of 20 nm, fluorophores must be placed and localized every 10 nm. For a 2D structure, a labeling density of approximately 10^4^ fluorophores μm^−2^ or approximately 600 fluorophores within a circular diffraction-limited region is required (Sauer 2013).

Thus, independent of the technique used, efficient and specific labeling with fluorescent probes is a decisive aspect of super-resolution imaging. Fluorescent proteins are the gold standard for stoichiometric labeling of proteins in living cells. However, they also exhibit a non-negligible size (a cylinder with a length of 4.2 nm and diameter of 2.4 nm) (Tsien 1998). Organic fluorophores such as rhodamine or carbocyanine dyes are substantially smaller (~1 nm) but require a chemical labeling procedure such as standard immunofluorescence using primary and secondary IgG antibodies with a size of ~10 nm (Weber et al. 1978). Alternatively, small labeled camelid antibodies (nanobodies) directed against green fluorescent protein (GFP) (Ries et al. 2012) with a smaller size (1.5 nm × 2.5 nm) can be used for efficient labeling. One of the most promising future labeling methods for high-density labeling is the 1,2,3-triazole linkage (click chemistry) between a fluorophore modified as an azide and a reaction partner (e.g., protein) modified as an alkyne, or vice versa (Laughlin et al. 2008). This method, however, requires further improvements concerning specific labeling of the protein of interest in living and fixed cells.

## Live-cell localization microscopy

Photoswitchable or photoactivatable fluorescent proteins (PA-FPs) were the first choice for super-resolution imaging in living cells, as they enable stoichiometric labeling and non-pertubative imaging of the sample (Lippincott-Schwartz and Patterson 2003). Several studies have been conducted using PA-FPs to unravel sub-diffractive morphologies and dynamics of cellular components (Table 2).

**Table 2:** Fluorophores used in live-cell single-molecule localization microscopy studies

	Fluorophore	λ_abs_/λ_em_ (nm)	Photons/on-event	Tag	Additives	Camera frame rate (Hz)	Temporal resolution (s)	Spatial resolution	References
Oregon green	Rhodamine	490/514	900	Snap	None	100–150	30	3D: 50 nm *xy*; 90 nm *z*	Jones et al. (2011)
Alexa 488	Rhodamine	496/520	400	Snap	Glutathione, oxygen scavenger	30	150	n/a	Benke et al. (2012)
PA-GFP^a^	FP	504/517	n/a	fusion protein	None	5–10	100–300	40 nm	Hess et al. (2007)
PA-GFP^a^	FP	504/517	n/a	fusion protein	None	30	90	25 nm	Wilmes et al. (2012)
505	Rhodamine	504/532	300–2,000	Snap	None	30–100	60–300	n/a	Klein et al. (2011)
EYFP	FP	514/527	741	fusion protein	None	10	60–120	40 nm	Biteen et al. (2008)
Dil	Carbocyanine	549/565	720	none	Oxygen scavenger	500	15	40 nm	Shim et al. (2012)
TMR	Rhodamine	554/580	1,100	Snap	None	100–150	30	3D: 45 nm *xy*; 82 nm *z*	Jones et al. (2011)
TMR	Rhodamine	554/580	300–2,000	Snap	None	30–100	60–300	n/a	Klein et al. (2011)
TMR	Rhodamine	554/580	n/a	Clip	Gluthatione, oxygen scavenger	30–50	200–350	n/a	Klein et al. (2012)
TMR	rhodamine	554/580	n/a	Halo	None	31	6–16	15 nm	Appelhans et al. (2012)
PATagRFP^a^	FP	562/595	n/a	fusion protein	None	30	90	25 nm	Wilmes et al. (2012)
tdEos^a^	FP	569/581	750	fusion protein	None	25	25–60	60 nm	Shroff et al. (2008)
tdEos^a^	FP	569/581	1,200	fusion protein	None	100–150	3D: 30	3D: 40 nm *xy*; 80 nm *z*	Jones et al. (2011)
EosFP^a^	FP	571/581	n/a	fusion protein	None	20	500	25 nm	Manley et al. (2008)
mEos2^a^	FP	573/584	1,200	fusion protein	None	100–150	3D: 30	3D: 40 nm *xy*; 80 nm *z*	Jones et al. (2011)
Alexa 568	Rhodamine	572/600	1,700	directly labeled	β-Mercaptoethanol, oxygen scavenger	500	3D: 30	3D: 40 nm *xy*; 70 nm *z*	Jones et al. (2011)
LysoTracker Red	Bodipy	577/590	820	none	Oxygen scavenger	500	1	30 nm	Shim et al. (2012)
MitoTracker Red	Cationic rosamine	581/644	790	none	Oxygen scavenger	500–900	2–10	30–40 nm	Shim et al. (2012)
ER-Tracker Red	Bodipy	587/615	820	none	Oxygen scavenger	500	10	35 nm	Shim et al. (2012)
Alexa 647	cyanine	649/670	3,500	directly labeled, Snap	β-Mercaptoethanol, oxygen scavenger	500	2D: 0.5 3D: 1–2	2D: 25 nm 3D: 30 nm *xy*; 50 nm *z*	Jones et al. (2011)
Alexa 647	Cyanine	649/670	n/a	Snap	Gluthatione, oxygen scavenger	30–50	200–350	n/a	Klein et al. (2012)
SiR	Silicon-rhodamine	650/668	630	Snap	None	50	200	n/a	Lukinavicius et al. (2013)
ATTO 655	Oxazine	663/684	500–1,500	TMP	None	50	10	20 nm	Wombacher et al. (2010)
ATTO 655	Oxazine	663/684	1,200	Snap	None	100–150	30	3D: 40 nm *xy*; 80 nm *z*	Jones et al. (2011)
ATTO 655	Oxazine	663/684	800	Halo	None	30	90	25 nm	Wilmes et al. (2012)

*FP* fluorescent protein

^a^converted/activated form

One of the first realizations of live-cell super-resolution imaging investigated the structure of the actin protein MreB labeled with EYFP by PALM (Biteen et al. 2008). Furthermore, they introduced time-lapse PALM to increase the effective labeling concentration and achieve a sub-40 nm spatial resolution. Images were obtained with cycles of reactivation at 407 nm and imaging of EYFP at 514 nm with a frame rate of 10 Hz and 1–2 min temporal resolution.

PALM was also successfully implemented to investigate the nanoscale dynamics of adhesion complexes by imaging paxillin fused to tdEosFP (Shroff et al. 2008). Since the dynamics of these complexes occurs at a timescale of minutes, a series of PALM images, acquired over a time >20 min, with a temporal resolution of 25–60 s was sufficient to study morphological changes. PALM imaging revealed sub-diffractive structures and different dynamics within an adhesion complex and between complexes at a spatial resolution of about 60 nm. Assembly and disassembly of adhesions in different cell lines were also studied. With FPALM imaging of PA-GFP, the motion of hemagglutinin (HA) clusters in the cell membrane of fibroblasts with a localization precision of ~40 nm was investigated (Hess et al. 2007) by calculating the distance distribution of HA molecules in consecutive frames with a time resolution of 100–300 s. Thus, an effective diffusion coefficient could be determined. A combination of single-particle tracking and PALM imaging (sptPALM) was introduced to study the mobility and distribution of two membrane proteins (VSVG and Gag) by Manley et al. (2008). The experiments gave single-molecule trajectories at a high density (50 μm^−2^) and the mapping of single-molecule diffusion coefficients of VSVG and Gag evidenced that VSVG proteins exhibit a larger mobile fraction than Gag proteins.

Due to the comparatively low fluorescence quantum yield and photostability of FPs, (F)PALM in living cells is restricted to slower cellular processes. Imaging faster dynamic processes in cells requires higher temporal resolution, ideally without decreasing the spatial resolution. In this regard, organic dyes are the fluorophores of choice. They emit more photons than FPs and show tunable photoswitching rates. Therefore, they are well suited for improving the spatiotemporal resolution in live-cell localization microscopy experiments.

Chemical tags (Gautier et al. 2008; Keppler et al. 2003; Los et al. 2008; Miller et al. 2005) offer an elegant method for specific labeling of proteins with organic dyes in living cells (Table 2). Here, a polypeptide tag, which is genetically fused to the target protein, binds to a fluorescently labeled substrate. On the other hand, live-cell labeling with chemical tags is also challenging because the fluorescently labeled substrates have to exhibit high membrane permeability and negligible non-specific binding in living cells. Thus, the labeling procedure has to be optimized for each protein and fluorescent substrate separately concerning substrate concentration and incubation time. Furthermore, the amount of non-specific binding of fluorescently substrates to cellular organelles has to be carefully checked. Nonetheless, some fluorescently labeled substrates are very well suited for specific labeling of proteins in living cells.

**Fig. 1:**
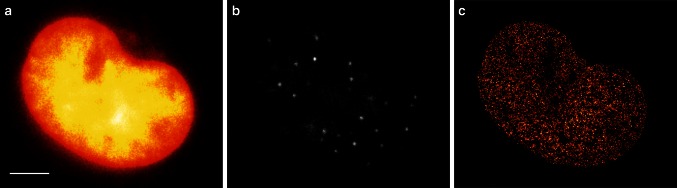
Live-cell *d*STORM with SNAP tags. **a** Fluorescence image of histone H2B proteins in a COS-7 cell stained with SNAP-Cell TMR-Star (1 μM). *Scale bar* 5 μm. **b** Fluorescence image of the same cell but with 532 nm excitation of ~1 kW cm^−2^, which induced photoswitching. **c** *d*STORM image reconstructed from 10,000 images (acquired at 50 Hz). Adapted from (Klein et al. 2011), with permission

The first demonstration of live-cell single-molecule localization microscopy with organic dyes was performed by *d*STORM employing the trimethoprim (TMP) tag (Wombacher et al. 2010). TMP binds to the *E. coli* dihydrofolate reductase (eDHFR). The cell-permeable dye ATTO 655 was used to label the eDHFR tagged core histone protein H2B. Since the cell provides its own reductive environment, mostly due to the presence of millimolar concentration of the thiol glutathione (van de Linde et al. 2012), ATTO 655 molecules switch between a bright on- and a non-fluorescent off-state upon irradiation under physiological conditions. Applying a sliding window analysis on the localization data, the dynamic movement of histone proteins could be observed at a temporal resolution of 10 s. Slightly later, the Snap-tag technology was also successfully applied for live-cell *d*STORM in various cell lines using the two rhodamine-based substrates SNAP-Cell TMR-Star and SNAP-Cell 505 (Fig. 1) (Klein et al. 2011). The results demonstrated the formation of long-lived fluorophore off-states under physiological conditions, which can be effectively and repeatedly reactivated upon irradiation at 405 nm (Klein et al. 2011; van de Linde et al. 2011a, b). Because rhodamine dyes tend to bind non-specifically on glass surfaces, coverslips were coated with 2 M glycine.

The photoswitchability of some organic fluorophores in living cells was also used to study the organization of intracellular microcompartments (Appelhans et al. 2012). Here, mitochondrial proteins of the outer and inner membrane were labeled with tetramethylrhodamine (TMR) via the HaloTag. Single-molecule tracking and localization of individual protein complexes showed protein-specific diffusion behavior within both membranes and their substructures like cristae, indicating mitochondrial compartmentalization.

As the different chemical tags can be used orthogonally, multi-color super-resolution imaging is likewise possible. For single-molecule tracking of two different plasma membrane receptor proteins, a multi-color approach employing Snap- and HaloTag has been demonstrated (Benke et al. 2012). The fluorophores used (Dy-547 for Snap and Alexa 488 for Halo) are spectrally well separated and were imaged sequentially. In addition, the study demonstrated that tracking is not restricted to membrane proteins, but is also applicable to intracellular proteins like H2B. Using a Snap- and Clip tag in combination with the two dyes Alexa 647 and TMR, dual-color live-cell *d*STORM of the beta-2-adrenergic receptor and H2B histone proteins has been demonstrated (Klein et al. 2012). The membrane receptor was labeled with the cell-impermeable dye Alexa 647 and the nucleus with TMR. To induce photoswitching of Alexa 647, the medium was supplemented with 100 mM glutathione and an oxygen scavenger system.

However, in all live-cell experiments using organic fluorophores, the generation of reactive oxygen species (ROS) has to be considered (van de Linde et al. 2011a). ROS may already perturb the intracellular environment during the experiment and thus can be expected to be a serious source of cell damage.

The possibility to combine PALM and *d*STORM for two-color super-resolution imaging was shown by Endesfelder et al. for fixed cells using the same buffer (Endesfelder et al. 2011). For live-cell imaging, the combination of PA-FPs and chemical tags enabled also three-color PALM/*d*STORM of the spatiotemporal organization of membrane receptors (Wilmes et al. 2012). Fusion proteins of the type I interferon receptors (IFNAR1 and IFNAR2) with PA-GFP and PAtagRFP were coexpressed with the actin-binding Lifeact peptide (Riedl et al. 2008) coupled to HaloTag, which was stained with ATTO 655. Three-color super-resolution imaging was performed through cycles of photoactivation at 405 nm and sequential excitation at 488 nm for PA-GFP, 568 nm for PAtagRFP, and 647 nm for ATTO 655. The average localization precision achieved in these experiments was ~25 nm. The reconstructed images showed a cytoskeletal association of the two receptors, indicating an interaction with actin or actin-bound adaptor proteins (Wilmes et al. 2012).

Small membrane probes pose a different tag-independent labeling approach for membrane structures. They bind directly to the membrane of mitochondria, endoplasmic reticulum, lysosomes, or the plasma membrane. These commercially available probes proved to be photoswitchable in living cells upon irradiation with the respective wavelength and reactivation at 405 nm (Shim et al. 2012). An oxygen scavenger system was applied to reduce photobleaching and prolong data acquisition time. Due to the high labeling density, the ultrastructural dynamics of organelles could be resolved with a spatial resolution of ~40 nm and temporal resolution of 1–15 s using multi-emitter fitting algorithms. Time-lapse imaging of mitochondria with MitoTracker Red, for example, revealed thin tubular intermediates between mitochondria at fission and fusion events, which could not be resolved in conventional diffraction-limited imaging (Shim et al. 2012).

Three-dimensional super-resolution imaging in live cells has also been demonstrated (Jones et al. 2011). Clathrin-coated pits and their cargo transferrin were labeled with different fluorophores using Snap-tag fusion proteins or direct labeling. Due to high laser intensities used of up to 15 kW cm^−2^ and the resulting fast switching kinetics of Alexa 647, a time resolution of 0.5 s and 25 nm spatial resolution was achieved in 2D and 2 s and 50 nm (axial) in 3D, respectively. Only with Alexa 647, which was delivered into the cell by electroporation, the cup-like morphology of CCPs could be resolved.

Very recently, a new near-infrared fluorophore has been introduced for live-cell imaging (Lukinavicius et al. 2013). This new silicon–rhodamine (SiR) is highly cell-permeable and shows a fivefold increase in fluorescence upon binding to its target protein. The fluorogenic character results from the formation of the non-fluorescent spirolactone if the dye aggregates or binds unspecifically to hydrophobic structures, whereas it becomes fluorescent upon reaction with the respective protein tag. Single-molecule localization microscopy is also feasible, as SiR exhibits switching behavior under physiological conditions, which was shown for cells expressing H2B–Snap (Lukinavicius et al. 2013).

## 3D super-resolution imaging

In order to enable three-dimensional (3D) super-resolution imaging, the axial symmetry of the PSF has to be broken, that is, in conventional microscopy (in the case of zero aberrations), the PSF of a fluorophore slightly above and below the image plane appears equally blurred preventing the extraction of the accurate axial fluorophore position. To overcome the obstacle methods have been introduced that tweak the signal in a way that the PSF above and below the image plane looks different. Some of the ideas have already been successfully used in single-particle tracking, and with the ability to image only a few fluorophores defining a densely labeled structure at a time due to temporal separation of fluorophore emission, it was straightforward to use these established methods also for localization microscopy. Methods used to infer information about the axial origin of a signal can roughly be split into four groups: (1) astigmatism, (2) biplane, (3) PSF splitting into lobes, and (4) interferometric approaches (Table 3).

**Table 3:** Three-dimensional single-molecule localization microscopy techniques

Technique	Lateral/axial resolution (FWHM) (nm)	Axial capture range	References
Astigmatism (polynomial)	20–30/60–70	0.6 μm; with stacking up to 3 μm	Huang et al. (2008a)
Astigmatism (polynomial, dual objective)	9/19	0.6 μm	Xu et al. (2012)
Astigmatism (sigma difference)^a^	40/50	1 μm	Henriques et al. (2010)
Astigmatism (model-free)^b^	<50/<100	≥3 μm	York et al. (2011)
Biplane	30/75	0.8 μm	Juette et al. (2008)
Double–helix PSF	30/46	>2 μm	Pavani et al. (2009)
PRILM	38–47/82	2 μm	Baddeley et al. (2011)
iPALM	22.8/9.8	0.225 μm	Shtengel et al. (2009)

^a^ *z*-position determination via sigma difference lookup table

^b^Direct *z*-position determination by comparison with experimental PSF

**Fig. 2:**
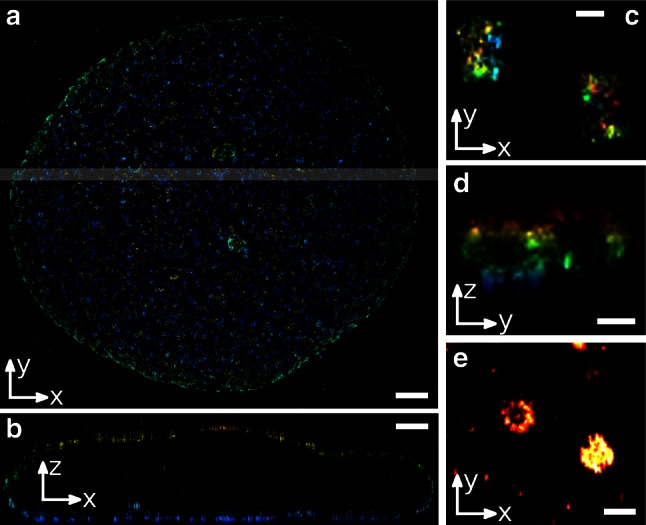
**a** Nucleus of a *Xenopus laevis* A6 cell stained against the nuclear pore complex protein gp210 with pale white bar indicating the area where the *x*–*z*-cross section **b** is taken; **c** and **d** show the respective *x*–*y*- and *y*–*z*-views of the distal appendage protein CEP152 of centrioles from a U2OS cell; **e** represents another pair of centrioles in a COS-7 cell. All stainings were performed with Alexa Fluor 647. *Scale bar* **a**, **b** 1 μm; **c**, **d** 200 nm; **e** 500 nm; color-code (*blue* to *red*) **a**, **b** 0–4.6 μm; **c**, **d** 0–400 nm

The astigmatism approach introduces a cylindrical lens into the detection path of the microscope system. This leads to a stretching of the PSF because only one spatial direction is tightly focused while the other is defocused (Fig. 2). In practice, there is one point of equal PSF widths (which can be set to be *z* = 0 nm), and PSFs originating from above or below are spread to a horizontal or vertical line, respectively. The rough axial position can be extracted from the orientation of the PSF and the widths in *x* and *y* can be calibrated to yield exact *z*-coordinates. After the initial use in single-particle tracking (Holtzer et al. 2007; Kao and Verkman 1994), the method has been broadly applied to single-molecule localization microscopy (Dani et al. 2010; Huang et al. 2008a, b; Xu et al. 2012). For calibration of the defocusing behavior, usually single fluorophores, quantum dots or small fluorescent beads are adsorbed on a bare coverslip and moved in *z* while recording their PSF. The obtained PSF widths in *x* and *y* are fitted with a polynomial of second order, which represents a physically derived model (Holtzer et al. 2007) or a fourth-order polynomial to account for imperfections in the optical system (Huang et al. 2008a). To avoid fitting of a more or less physically derived function to the calibration data, a lookup table can be created for the extraction of the actual axial position. In the open-source QuickPALM plugin for ImageJ (Henriques et al. 2010), the standard deviations of the calibration PSF in *x* and *y* are determined and the known *z*-position is plotted against *σ*_*x*_ − *σ*_*y*_. The obtained straight line serves to look up the *z*-coordinates during the course of a measurement.

To use biplane imaging, the detected signal has to be split into two parts with an optical delay added to one of the two signals. Both signal paths can be either imaged on the same camera (Juette et al. 2008), or if a second camera is at hand, the two signals can be detected separately. In the second scheme, the full field of view is preserved. In both approaches, a relative offset in the axial position between both image planes has to be realized. The axial position can be extracted from the sharpness ratio of the respective planes. The PSF widths detected on detector A and B encode the *z*-coordinate just as the ratio between the *x-* and *y*-PSF widths using the astigmatism approach.

The third family of 3D localization microscopy techniques uses additional optics to split the PSF of a fluorophore into two lobes that rotate around each other with respect to the axial position of the emitter. It was first proposed to engineer the PSF to a double-helix by the introduction of a phase mask, which is created by a polarization-sensitive spatial light modulator (SLM) (Pavani et al. 2009). The two resulting lobes rotate around each other in a double-helix (DH-PSF) manner, which baptized the technique. It was later proposed to use a simplified approach adding a phase ramp in the objective aperture to half of the signal (Baddeley et al. 2011) instead of a SLM in the light path. In this method, termed phase ramp imaging localization microscopy (PRILM), in contrast to DH-PSF, one lobe remains fixed while the other rotates around it. Both techniques have in common that the available axial range is significantly larger than in biplane- or astigmatic imaging (Baddeley et al. 2011).

In the interferometric iPALM approach, the sample is placed between two objectives, and thus, the signal of a single fluorophore travels along two different light paths. Exploiting the wave–particle duality, upon superposition of the respective signals with a three-way beam splitter, every single photon will interfere with itself. Depending on the axial position, i.e., whether it is closer to objective A or B, the fluorophore will show distinct interference that gives information about the *z*-coordinate (Kanchanawong et al. 2010; Shtengel et al. 2009). In good agreement with the sub-wavelength sensitivity, interferometry so far yields the best axial localization precision but is limited in axial range to about half the emitted wavelength (Table 3).

## Technical challenges of 3D localization microscopy

Most 3D localization microscopy approaches use high NA oil-immersion objectives for surface confined total internal reflection fluorescence (TIRF) irradiation. Another very popular irradiation mode for intracellular imaging only available with oil immersion is highly inclined and laminated optical sheet (HILO) microscopy (Tokunaga et al. 2008).

In a sense, HILO is similar to TIRF, where illuminating light is approaching the sample at a critical angle, so that only molecules very close to the coverslip are illuminated by the evanescent wave. However, instead of illuminating only the molecules in close proximity to the coverslip as in TIRF, HILO illuminates by a highly inclined and thin beam under a particular angle allowing high irradiation intensities combined with high signal-to-noise ratio even inside of cells. Unfortunately, using an oil-immersion lens for imaging while measuring in aqueous buffer induces aberrations due to the refractive-index mismatch at the coverslip–buffer interface. Thus, the volume image of a specimen will appear stretched along the optical axis, an effect well described for both confocal (Hell et al. 1993) and single-molecule localization microscopy. The image stretching can either be accounted for experimentally (Huang et al. 2008b) or analytically by rescaling with the factor *η*_buffer_/*η*_immersion_ (Biteen et al. 2012).

Furthermore, the refractive-index mismatch leads to spherical aberration along the *z*-axis that is difficult to correct (Deng and Shaevitz 2009). By localizing a single fluorescent particle placed in various but well-controlled depths by an optical tweezer, it was shown that the determined *z*-position could be up to 30 % erroneous. This issue is especially tricky, as the amount of false localization is not uniform along the *z*-axis, that is, if single fluorophores adsorbed on a bare glass surface were used for calibration and the subsequent measurement is taken slightly above the surface, the localization determination between coverslip and focal plane will perform precisely, while a systematic false coordinate determination will occur for fluorophores above the focal plane.

Both sorts of aberrations can be avoided by either adjusting the refractive index of the imaging buffer (Huang et al. 2008a) or by using objectives that use immersion media with matching refractive index (glycerol immersion for tissue imaging or water immersion for adherent cells). However, the resulting epifluorescence illumination geometry results in lower excitation intensities, higher background, and increased photodamage rendering live-cell super-resolution imaging experiments more difficult. In addition, when working with glycerol and water objectives, it is crucially important to ensure that the sample is aligned perfectly horizontally. It was shown that even small tilt angles of less than 1° deteriorate the concentric pattern of the PSF (Arimoto and Murray 2004). The center of a PSF above or below the focal plane will thus appear to be shifted to the same side, which is a nice indication for coverslip tilt if encountered. Please note that this aberration virtually does not occur for oil-immersion objectives. Thus, it has to be affirmed that the stage is perfectly leveled before the immersion medium is changed.

Finally, it has to be considered that the time needed to fully image a large 3D volume is significantly higher compared to one single 2D snapshot because several layers have to be imaged. Unfortunately, with longer imaging times, setups are more prone to drift, which causes severe difficulties for later image processing. Drift compensation is usually performed by fluorescence tracking of non-blinking fiducials. Using a hydrogel with a refractive index close to water, 100 nm fiducials can be stably immobilized and homogeneously distributed in 3D even in close vicinity of cells (Zessin et al. 2013). In addition, the fiducials can be used advantageously for multicolor alignment.

## Super-resolution imaging of tissue and organs

**Fig. 3:**
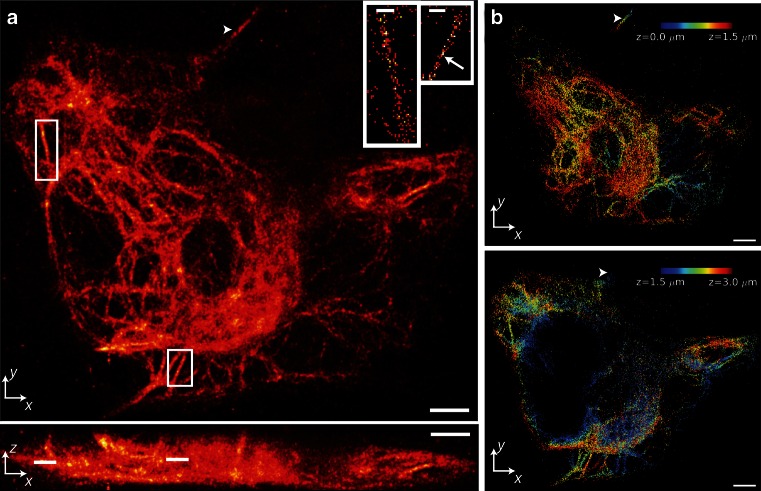
Three-dimensional PALM imaging of a vimentin network. **a** *xy* (*top*) and *xz* (*bottom*) maximum-intensity projections of PA-mCherry1–vimentin. About 1 million unlinked localizations was rendered in each view.* Insets* show further magnification of white rectangles in *xy* (lines in *xz*) maximum-intensity projection, highlighting individual vimentin fibrils in 60-nm-thick *z*-slices (localizations are linked). *Arrow* region of fibril with apparent width <100 nm. **b** Axial extent of vimentin network with *z*-location indicated as a color map. For clarity, localizations corresponding to 0–1.5 μm (*top*) and 1.5–3 μm (*bottom*) are shown separately. *Arrowheads* in *a* and *b* indicate a fibril that persists over >2 μm axially. Only linked localizations with correlation strength >0.4 are shown. Histogram bin sizes are 60 nm for all subfigures. *Scale bars* 3 μm (**a**), 600 nm (*insets*), 3 μm (**b**). Reproduced from York et al. (2011), with permission

In order to enable super-resolution imaging of whole living organisms, novel long-term continuous imaging methods with high spatial resolution and minimal photodamage such as selective plane illumination microscopy (SPIM) (Huisken et al. 2004) or Bessel beam plane illumination (Gao et al. 2012; Planchon et al. 2011) are urgently needed. Ideally, these methods are combined with the enhanced penetration depth and axial sectioning capabilities of two-photon microscopy. First, realizations of super-resolution imaging with confined two-photon activation (York et al. 2011) (Fig. 3) and SPIM (Cella Zanacchi et al. 2011, 2013) have recently been demonstrated. Besides the use of photoactivatable fluorescent proteins, so-called caged, non-fluorescent organic dyes that can be activated upon irradiation with UV or IR light, such as rhodamine NN dyes (Belov et al. 2010) and carborhodamine dyes masked with photolabile groups (Grimm et al. 2013), could also be used advantageously for axially confined localization microscopy.

Alternatively, photoswitchable fluorescent proteins and superimposed orthogonal standing light waves generating thousands of doughnuts can be used for live-cell super-resolution imaging according to the RESOLFT principle (standing for reversible saturable optical fluorescence transitions). Relying on the use of fluorescent proteins, which can be photoswitched between a stable on- and off-state under substantially lower light intensities than STED microscopy, the method enables super-resolution imaging of large field of views (>100 μm × 100 μm) in less than 1 s with subdiffraction spatial resolution (Chmyrov et al. 2013).

For sensitive fluorescence imaging of fixed whole organs, new sample preparation methods such as CLARITY (Chung and Deisseroth 2013) and 3DISCO (Ertürk et al. 2012) have recently been developed that make organs transparent to light while keeping them intact, providing a detailed glimpse of their inner structure. For example, CLARITY works by removing the fatty tissue that surrounds cells and makes them opaque, while preserving the tissue’s structure. Importantly, the tissue can be labeled with different fluorescent probes after a washing step. The potential of the technique has been demonstrated by imaging the brain of an adult mouse allowing the visualization of neuronal connections and local circuitry deep inside the brain on a cellular level (Chung and Deisseroth 2013). Congruously, the next step will be the visualization of neuronal connections inside the brain with molecular resolution applying the above-mentioned method.

## Conclusion and perspective

Single-molecule localization microscopy techniques have the potential to substantially improve our understanding of how cellular structure and function is organized. Currently, they cannot compete with deterministic super-resolution imaging techniques such as STED and SIM regarding imaging speed and temporal resolution in live-cell experiments, but they can provide single-molecule information about molecular distributions, even giving absolute numbers of proteins present in subcellular compartments. This ability provides the fascinating possibility to study encoding of cellular function at the molecular level.

To produce faster image readout speeds over large fields of view, Huang et al. used next-generation scientific CMOS camera technology in combination with localization microscopy (Huang et al. 2013). This enabled an imaging speed of up to 32 frames per second applying an efficient noise correction algorithm. However, with increasing imaging speed, the photon output of fluorophores also has to be improved. Therefore, new methods have to be developed that increase the localization precision by collecting more fluorescence photons per on-event. This can be achieved for example using two opposing objective lenses (Ram et al. 2009; Shtengel et al. 2009; Xu et al. 2012). Alternatively, the photoswitching rates can be decelerated by chemical or physical means, allowing the detection of more fluorescence photons per switching event (Sauer 2013). For example, triplet-state quenchers such as cyclooctatetraene (COT) (Altman et al. 2012) can be used to increase the number of fluorescence photons emitted per switching event by optimizing the cycling between the singlet and triplet states before the fluorophore eventually enters a long-lived off-state (Olivier et al. 2013). For some red-absorbing dyes, the fluorescence quantum yield and lifetime could be substantially increased exchanging water by heavy water (D_2_O) (Lee et al. 2013). The magnitude of this effect scales with the emission wavelength, reaching a particularly high value of 2.63-fold for the cyanine dye Cy7 (Klehs et al. 2013).

Alternatively, the already mentioned caged non-fluorescent organic dyes that can be activated upon irradiation with UV or IR light (Belov et al. 2010; Grimm et al. 2013) could be used in combination with a reducing and oxidizing system (ROXS), e.g., of 1 mM ascorbic acid and 1 mM methyl viologen under oxygen depletion (Vogelsang et al. 2008). ROXS increases the photostability of fluorophores by efficient depopulation of the triplet state and recovery of the singlet state (Vogelsang et al. 2008). Upon uncaging fluorophores emit more photons, thus improving the localization precision.

## References

[CR1] Abbe E (1873). Theorie des Mikroskops und der mikroskopischen Wahrnehmung. Arch Mikrosk Anat.

[CR2] Adams RL, Wente SR (2013). Uncovering nuclear pore complexity with innovation. Cell.

[CR3] Altman RB, Terry DS, Zhou Z, Zheng Q, Geggier P, Kolster RA, Zhao Y, Javitch JA, Warren JD, Blanchard SC (2012). Cyanine fluorophore derivatives with enhanced photostability. Nat Methods.

[CR4] Appelhans T, Richter CP, Wilkens V, Hess ST, Piehler J, Busch KB (2012). Nanoscale organization of mitochondrial microcompartments revealed by combining tracking and localization microscopy. Nano Lett.

[CR5] Arimoto R, Murray JM (2004). A common aberration with water-immersion objective lenses. J Microsc.

[CR6] Baddeley D, Jayasinghe ID, Cremer C, Cannell MB, Soeller C (2009). Light-induced dark states of organic fluochromes enable 30 nm resolution imaging in standard media. Biophys J.

[CR7] Baddeley D, Cannell M, Soeller C (2011). Three-dimensional sub-100 nm super-resolution imaging of biological samples using a phase ramp in the objective pupil. Nano Res.

[CR8] Bates M, Blosser TR, Zhuang XW (2005) Short-range spectroscopic ruler based on a single-molecule optical switch. Phys Rev Lett 94:108101 10.1103/PhysRevLett.94.108101 PMC2652517 15783528

[CR9] Bates M, Huang B, Dempsey GT, Zhuang X (2007). Multicolor super-resolution imaging with photo-switchable fluorescent probes. Science.

[CR10] Belov VN, Wurm CA, Boyarskiy VP, Jakobs S, Hell SW (2010). Rhodamines NN: a novel class of caged fluorescent dyes. Angew Chem Int Ed Engl.

[CR11] Benke A, Olivier N, Gunzenhauser J, Manley S (2012). Multicolor single molecule tracking of stochastically active synthetic dyes. Nano Lett.

[CR12] Betzig E (1995). Proposed method for molecular optical imaging. Opt Lett.

[CR13] Betzig E, Patterson GH, Sougrat R, Lindwasser OW, Olenych S, Bonifacino JS, Davidson MW, Lippincott-Schwartz J, Hess HF (2006). Imaging intracellular fluorescent proteins at nanometer resolution. Science.

[CR14] Biteen JS, Thompson MA, Tselentis NK, Bowman GR, Shapiro L, Moerner WE (2008). Super-resolution imaging in live Caulobacter crescentus cells using photoswitchable EYFP. Nat Methods.

[CR15] Biteen JS, Goley ED, Shapiro L, Moerner WE (2012). Three-dimensional super-resolution imaging of the midplane protein FtsZ in live Caulobacter crescentus cells using astigmatism. ChemPhysChem.

[CR16] Bock H, Geisler C, Wurm CA, von Middendorff C, Jakobs S, Schönle A, Egner A, Hell SW, Eggeling C (2007). Two-color far-field fluorescence nanoscopy based on photoswitchable emitters. Appl Phys B.

[CR17] Cella Zanacchi F, Lavagnino Z, Perrone Donnorso M, Del Bue A, Furia L, Faretta M, Diaspro A (2011). Live-cell 3D super-resolution imaging in thick biological samples. Nat Methods.

[CR18] Cella Zanacchi F, Lavagnino Z, Faretta M, Furia L, Diaspro A (2013). Light-sheet confined super-resolution using two-photon photoactivation. PLoS ONE.

[CR19] Chmyrov A, Keller J, Grotjohann T, Ratz M, d’Este E, Jakobs S, Eggeling C, Hell SW (2013). Nanoscopy with more than 100,000 ‘doughnuts’. Nat Methods.

[CR20] Chung K, Deisseroth K (2013). CLARITY for mapping the nervous system. Nat Method.

[CR21] Dani A, Huang B, Bergan J, Dulac C, Zhuang X (2010). Superresolution imaging of chemical synapses in the brain. Neuron.

[CR22] Dempsey GT, Bates M, Kowtoniuk WE, Liu DR, Tsien RY, Zhuang X (2009). Photoswitching mechanism of cyanine dyes. J Am Chem Soc.

[CR23] Dempsey GT, Vaughan JC, Chen KH, Bates M, Zhuang X (2011). Evaluation of fluorophores for optimal performance in localization-based super-resolution imaging. Nat Methods.

[CR24] Deng Y, Shaevitz JW (2009). Effect of aberration on height calibration in three-dimensional localization-based microscopy and particle tracking. Appl Opt.

[CR25] Dyba M, Hell SW (2002). Focal spots of size λ/23 open up far-field florescence microscopy at 33 nm axial resolution. Phys Rev Lett.

[CR26] Endesfelder U, van de Linde S, Wolter S, Sauer M, Heilemann M (2010). Subdiffraction-resolution fluorescence microscopy of myosin-actin motility. ChemPhysChem.

[CR27] Endesfelder U, Malkusch S, Flottmann B, Mondry J, Liguzinski P, Verveer PJ, Heilemann M (2011). Chemically induced photoswitching of fluorescent probes—a general concept for super-resolution microscopy. Molecules.

[CR28] Ertürk A, Becker K, Jährling N, Mauch CP, Hojer CD, Egen JG, Hellal F, Bradke F, Sheng M, Dodt HU (2012). Three-dimensional imaging of solvent-cleared organs using 3DISCO. Nat Protoc.

[CR29] Fölling J, Bossi M, Bock H, Medda R, Wurm CA, Hein B, Jakobs S, Eggeling C, Hell SW (2008). Fluorescence nanoscopy by ground-state depletion and single-molecule return. Nat Methods.

[CR30] Gao L, Shao L, Higgins CD, Poulton JS, Peifer M, Davidson MW, Wu X, Goldstein B, Betzig E (2012). Noninvasive imaging beyond the diffraction limit of 3D dynamics in thickly fluorescent specimens. Cell.

[CR31] Gautier A, Juillerat A, Heinis C, Corrêa IR, Kindermann M, Beaufils F, Johnsson K (2008). An engineered protein tag for multiprotein labeling in living cells. Chem Biol.

[CR32] Geisler C, Schönle A, von Middendorff C, Bock H, Eggeling C, Egner A, Hell SW (2007). Resolution of λ/10 in fluorescence microscopy using fast single molecule photo-switching. Appl Phys A.

[CR33] Giannone G, Hosy E, Levet F, Constals A, Schulze K, Sobolevsky AI, Rosconi MP, Gouaux E, Tampe R, Choquet D, Cognet L (2010). Dynamic superresolution imaging of endogenous proteins on living cells at ultra-high density. Biophys J.

[CR34] Giepmans BNG, Adams SR, Ellisman MH, Tsien RY (2006). The fluorescent toolbox for assessing protein location and function. Science.

[CR35] Grimm JB, Sung AJ, Legant WR, Hulamm P, Matlosz SM, Betzig E, Lavis LD (2013). Carbofluoresceins and carborhodamines as scaffolds for high-contrast fluorogenic probes. ACS Chem Biol.

[CR36] Gunkel M, Erdel F, Rippe K, Lemmer P, Kaufmann R, Hormann C, Amberger R, Cremer C (2009). Dual color localization microscopy of cellular nanostructures. Biotechnol J.

[CR37] Gustafsson MG (2000). Surpassing the lateral resolution limit by a factor of two using structured illumination microscopy. J Microsc.

[CR38] Gustafsson MG (2005). Nonlinear structured-illumination microscopy: wide-field fluorescence imaging with theoretically unlimited resolution. Proc Natl Acad Sci USA.

[CR39] Haugland RP (2005). The handbook—a guide to fluorescent probes and labeling technologies.

[CR40] Heilemann M, Herten DP, Heintzmann R, Cremer C, Müller C, Tinnefeld P, Weston KD, Wolfrum J, Sauer M (2002). High-resolution colocalization of single dye molecules by fluorescence lifetime imaging microscopy. Anal Chem.

[CR41] Heilemann M, Margeat E, Kasper R, Sauer M, Tinnefeld P (2005). Carbocyanine dyes as efficient reversible single-molecule optical switch. J Am Chem Soc.

[CR42] Heilemann M, van de Linde S, Schuttpelz M, Kasper R, Seefeldt B, Mukherjee A, Tinnefeld P, Sauer M (2008). Subdiffraction-resolution fluorescence imaging with conventional fluorescent probes. Angew Chem Int Ed Engl.

[CR43] Heilemann M, van de Linde S, Mukherjee A, Sauer M (2009). Super-resolution imaging with small organic fluorophores. Angew Chem Int Ed Engl.

[CR44] Heintzmann R, Cremer CG (1999) Laterally modulated excitation microscopy: improvement of resolution by using a diffraction grating. Vol Proc SPIE3568 185–196

[CR45] Hell SW (2009). Microscopy and its focal switch. Nat Methods.

[CR46] Hell SW, Wichmann J (1994). Breaking the diffraction resolution limit by stimulated emission: stimulated-emission-depletion fluorescence microscopy. Opt Lett.

[CR47] Hell S, Reiner G, Cremer C, Stelzer EHK (1993). Aberrations in confocal fluorescence microscopy induced by mismatches in refractive index. J Microsc.

[CR48] Henriques R, Lelek M, Fornasiero EF, Valtorta F, Zimmer C, Mhlanga MM (2010). QuickPALM: 3D real-time photoactivation nanoscopy image processing in ImageJ. Nat Methods.

[CR49] Hess ST, Girirajan TP, Mason MD (2006). Ultra-high resolution imaging by fluorescence photoactivation localization microscopy. Biophys J.

[CR50] Hess ST, Gould TJ, Gudheti MV, Maas SA, Mills KD, Zimmerberg J (2007). Dynamic clustered distribution of hemagglutinin resolved at 40 nm in living cell membranes discriminates between raft theories. Proc Natl Acad Sci USA.

[CR51] Holtzer L, Meckel T, Schmidt T (2007). Nanometric three-dimensional tracking of individual quantum dots in cells. Appl Phys Lett.

[CR52] Huang B, Jones SA, Brandenburg B, Zhuang X (2008). Whole-cell 3D STORM reveals interactions between cellular structures with nanometer-scale resolution. Nat Methods.

[CR53] Huang B, Wang WQ, Bates M, Zhuang XW (2008). Three-dimensional super-resolution imaging by stochastic optical reconstruction microscopy. Science.

[CR54] Huang F, Hartwich TMP, Rivera-Molina FE, Lin Y, Duim WC, Long JJ, Uchil PD, Myers JR, Baird MA, Mothes W, Davidson MW, Toomre D, Bewersdorf J (2013). Video-rate nanoscopy using sCMOS camera-specific single-molecule localization algorithms. Nat Methods.

[CR55] Huisken J, Swoger J, Del Bene F, Wittbrodt J, Stelzer EH (2004). Optical sectioning deep inside live embryos by selective plane illumination microscopy. Science.

[CR56] Jones SA, Shim SH, He J, Zhuang X (2011). Fast, three-dimensional super-resolution imaging of live cells. Nat Methods.

[CR57] Juette MF, Gould TJ, Lessard MD, Mlodzianoski MJ, Nagpure BS, Bennett BT, Hess ST, Bewersdorf J (2008). Three-dimensional sub-100 nm resolution fluorescence microscopy of thick samples. Nat Methods.

[CR58] Kaminski Schierle GS, van de Linde S, Erdelyi M, Esbjörner EK, Klein T, Rees E, Bertoncini CW, Dobson CM, Sauer M, Kaminski CF (2011). In situ measurements of the formation and morphology of intracellular β-amyloid fibrils by super-resolution fluorescence imaging. J Am Chem Soc.

[CR59] Kanchanawong P, Shtengel G, Pasapera AM, Ramko EB, Davidson MW, Hess HF, Waterman CM (2010). Nanoscale architecture of integrin-based cell adhesions. Nature.

[CR60] Kao HP, Verkman AS (1994). Tracking of single fluorescent particles in three dimensions: use of cylindrical optics to encode particle position. Biophys J.

[CR61] Keppler A, Gendreizig S, Gronemeyer T, Pick H, Vogel H, Johnsson K (2003). A general method for the covalent labeling of fusion proteins with small molecules in vivo. Nat Biotechnol.

[CR62] Klehs K, Spahn C, Endesfelder U, Lee SF, Fürstenberg A, Heilemann M (2013) Increasing the brightness of cyanine fluorophores for single-molecule and superresolution imaging. Chem Phys Chem. doi:10.1002/cphc.201300874 10.1002/cphc.201300874 24376142

[CR63] Klein T, Löschberger A, Proppert S, Wolter S, van de Linde S, Sauer M (2011). Live-cell dSTORM with SNAP-tag fusion proteins. Nat Methods.

[CR64] Klein T, van de Linde S, Sauer M (2012). Live-cell super-resolution imaging goes multicolor. ChemBioChem.

[CR65] Kner P, Chhun BB, Griffis ER, Winoto L, Gustafsson MG (2009). Super-resolution video microscopy of live cells by structured illumination. Nat Methods.

[CR66] Kuno M, Fromm DP, Hamann HF, Gallagher A, Nesbitt DJ (2000). Nonexponential “blinking” kinetics of single CdSe quantum dots: a universal power law behavior. J Chem Phys.

[CR67] Lampe A, Haucke V, Sigrist SJ, Heilemann M, Schmoranzer J (2012). Multi-colour direct STORM with red emitting carbocyanines. Biol Cell.

[CR68] Laughlin ST, Baskin JM, Amacher SL, Bertozzi CR (2008). In vivo imaging of membrane-associated glycans in developing zebrafish. Science.

[CR69] Lee SF, Vérolet Q, Fürstenberg A (2013). Improved super-resolution microscopy with oxazine fluorophores in heavy water. Angew Chem Int Ed.

[CR70] Lemmer P, Gunkel M, Baddeley D, Kaufmann R, Urich A, Weiland Y, Reymann J, Muller P, Hausmann M, Cremer C (2008). SPDM: light microscopy with single-molecule resolution at the nanoscale. Appl Phys B Lasers Opt.

[CR71] Lidke K, Rieger B, Jovin T, Heintzmann R (2005). Superresolution by localization of quantum dots using blinking statistics. Opt Express.

[CR72] Lippincott-Schwartz J, Patterson GH (2003). Development and use of fluorescent protein markers in living cells. Science.

[CR73] Lippincott-Schwartz J, Patterson GH (2009). Photoactivatable fluorescent proteins for diffraction-limited and super-resolution imaging. Trends Cell Biol.

[CR74] Los GV, Encell LP, McDougall MG, Hartzell DD, Karassina N, Zimprich C, Wood MG, Learish R, Ohana RF, Urh M, Simpson D, Mendez J, Zimmerman K, Otto P, Vidugiris G, Zhu J, Darzins A, Klaubert DH, Bulleit RF, Wood KV (2008). HaloTag: a novel protein labeling technology for cell imaging and protein analysis. ACS Chem Biol.

[CR75] Löschberger A, van de Linde S, Dabauvalle MC, Rieger B, Heilemann M, Krohne G, Sauer M (2012). Super-resolution imaging visualizes the eightfold symmetry of gp210 proteins around the nuclear pore complex and resolves the central channel with nanometer resolution. J Cell Sci.

[CR76] Lukinavicius G, Umezawa K, Olivier N, Honigmann A, Yang G, Plass T, Mueller V, Reymond L, Corrêa IR, Luo ZG, Schultz C, Lemke EA, Heppenstall P, Eggeling C, Manley S, Johnsson K (2013). A near-infrared fluorophore for live-cell super-resolution microscopy of cellular proteins. Nat Chem.

[CR77] Manley S, Gillette JM, Patterson GH, Shroff H, Hess HF, Betzig E, Lippincott-Schwartz J (2008). High-density mapping of single-molecule trajectories with photoactivated localization microscopy. Nat Methods.

[CR78] Mattila PK, Feest C, Depoil D, Treanor B, Montaner B, Otipoby Kevin L, Carter R, Justement Louis B, Bruckbauer A, Batista Facundo D (2013). The actin and tetraspanin networks organize receptor nanoclusters to regulate B cell receptor-mediated signaling. Immunity.

[CR79] Miller LW, Cai Y, Sheetz MP, Cornish VW (2005). In vivo protein labeling with trimethoprim conjugates: a flexible chemical tag. Nat Methods.

[CR80] Mortensen KI, Churchman LS, Spudich JA, Flyvbjerg H (2010). Optimized localization analysis for single-molecule tracking and super-resolution microscopy. Nat Methods.

[CR81] Olivier N, Keller D, Gönczy P, Manley S (2013). Resolution doubling in 3D-STORM imaging through improved buffers. PLoS ONE.

[CR82] Pavani SR, Thompson MA, Biteen JS, Lord SJ, Liu N, Twieg RJ, Piestun R, Moerner WE (2009). Three-dimensional, single-molecule fluorescence imaging beyond the diffraction limit by using a double-helix point spread function. Proc Natl Acad Sci USA.

[CR83] Pawley JB (2006). Handbook of biological confocal microscopy.

[CR84] Planchon TA, Gao L, Milkie DE, Davidson MW, Galbraith JA, Galbraith CG, Betzig E (2011). Rapid three-dimensional isotropic imaging of living cells using Bessel beam plane illumination. Nat Methods.

[CR85] Ram S, Prabhat P, Ward ES, Ober RJ (2009). Improved single particle localization accuracy with dual objective multifocal plane microscopy. Opt Express.

[CR86] Rayleigh L (1903). On the theory of optical images, with special reference to the microscope. J R Microsc Soc.

[CR87] Riedl J, Crevenna AH, Kessenbrock K, Yu JH, Neukirchen D, Bista M, Bradke F, Jenne D, Holak TA, Werb Z, Sixt M, Wedlich-Soldner R (2008). Lifeact: a versatile marker to visualize F-actin. Nat Methods.

[CR88] Ries J, Kaplan C, Platonova E, Eghlidi H, Ewers H (2012). A simple, versatile method for GFP-based super-resolution microscopy via nanobodies. Nat Methods.

[CR89] Roeffaers MBJ, De Cremer G, Libeert J, Ameloot R, Dedecker P, Bons A-J, Bückins M, Martens JA, Sels BF, De Vos DE, Hofkens J (2009). Super-resolution reactivity mapping of nanostructured catalyst particles. Angew Chem Int Ed.

[CR90] Rossy J, Owen DM, Williamson DJ, Yang Z, Gaus K (2013). Conformational states of the kinase Lck regulate clustering in early T cell signaling. Nat Immunol.

[CR91] Rust MJ, Bates M, Zhuang XW (2006). Sub-diffraction-limit imaging by stochastic optical reconstruction microscopy (STORM). Nat Methods.

[CR92] Sauer M (2013). Localization microscopy coming of age: from concepts to biological impact. J Cell Sci.

[CR93] Shannon CE (1949). Communication in the presence of noise. Proc IRE.

[CR94] Sharonov A, Hochstrasser RM (2006). Wide-field subdiffraction imaging by accumulated binding of diffusing probes. Proc Natl Acad Sci USA.

[CR95] Shim SH, Xia C, Zhong G, Babcock HP, Vaughan JC, Huang B, Wang X, Xu C, Bi GQ, Zhuang X (2012). Super-resolution fluorescence imaging of organelles in live cells with photoswitchable membrane probes. Proc Natl Acad Sci USA.

[CR96] Shroff H, Galbraith CG, Galbraith JA, White H, Gillette J, Olenych S, Davidson MW, Betzig E (2007). Dual-color superresolution imaging of genetically expressed probes within individual adhesion complexes. Proc Natl Acad Sci USA.

[CR97] Shroff H, Galbraith CG, Galbraith JA, Betzig E (2008). Live-cell photoactivated localization microscopy of nanoscale adhesion dynamics. Nat Methods.

[CR98] Shtengel G, Galbraith JA, Galbraith CG, Lippincott-Schwartz J, Gillette JM, Manley S, Sougrat R, Waterman CM, Kanchanawong P, Davidson MW, Fetter RD, Hess HF (2009). Interferometric fluorescent super-resolution microscopy resolves 3D cellular ultrastructure. Proc Natl Acad Sci USA.

[CR99] Sillibourne JE, Specht CG, Izeddin I, Hurbain I, Tran P, Triller A, Darzacq X, Dahan M, Bornens M (2011). Assessing the localization of centrosomal proteins by PALM/STORM nanoscopy. Cytoskeleton.

[CR100] Steinhauer C, Forthmann C, Vogelsang J, Tinnefeld P (2008). Superresolution microscopy on the basis of engineered dark states. J Am Chem Soc.

[CR101] Szymborska A, de Marco A, Daigle N, Cordes VC, Briggs JAG, Ellenberg J (2013). Nuclear pore scaffold structure analyzed by super-resolution microscopy and particle averaging. Science.

[CR102] Thompson RE, Larson DR, Webb WW (2002). Precise nanometer localization analysis for individual fluorescent probes. Biophys J.

[CR103] Tokunaga M, Imamoto N, Sakata-Sogawa K (2008). Highly inclined thin illumination enables clear single-molecule imaging in cells. Nat Methods.

[CR104] Tsien RY (1998). The green fluorescent protein. Annu Rev Biochem.

[CR105] van de Linde S, Kasper R, Heilemann M, Sauer M (2008). Photoswitching microscopy with standard fluorophores. Appl Phys B Lasers Opt.

[CR106] van de Linde S, Endesfelder U, Mukherjee A, Schüttpelz M, Wiebusch G, Wolter S, Heilemann M, Sauer M (2009). Multicolor photoswitching microscopy for subdiffraction-resolution fluorescence imaging. Photochem Photobiol Sci.

[CR107] van de Linde S, Krstic I, Prisner T, Doose S, Heilemann M, Sauer M (2011). Photoinduced formation of reversible dye radicals and their impact on super-resolution imaging. Photochem Photobiol Sci.

[CR108] van de Linde S, Löschberger A, Klein T, Heidbreder M, Wolter S, Heilemann M, Sauer M (2011). Direct stochastic optical reconstruction microscopy with standard fluorescent probes. Nat Protoc.

[CR109] van de Linde S, Heilemann M, Sauer M (2012). Live-cell super-resolution imaging with synthetic fluorophores. Annu Rev Phys Chem.

[CR110] van de Linde S, Aufmkolk S, Franke C, Holm T, Klein T, Löschberger A, Proppert S, Wolter S, Sauer M (2013). Investigating cellular structures at the nanoscale with organic fluorophores. Chem Biol.

[CR111] van Oijen AM, Köhler J, Schmidt J, Müller M, Brakenhoff GJ (1998). 3-Dimensional super-resolution by spectrally selective imaging. Chem Phys Lett.

[CR112] Vogelsang J, Kasper R, Steinhauer C, Person B, Heilemann M, Sauer M, Tinnefeld P (2008). A reducing and oxidizing system minimizes photobleaching and blinking of fluorescent dyes. Angew Chem Int Ed Engl.

[CR113] Vogelsang J, Cordes T, Forthmann C, Steinhauer C, Tinnefeld P (2009). Controlling the fluorescence of ordinary oxazine dyes for single-molecule switching and superresolution microscopy. Proc Natl Acad Sci USA.

[CR114] Weber K, Rathke PC, Osborn M (1978). Cytoplasmic microtubular images in glutaraldehyde-fixed tissue culture cells by electron microscopy and by immunofluorescence microscopy. Proc Natl Acad Sci USA.

[CR115] Williamson DJ, Owen DM, Rossy J, Magenau A, Wehrmann M, Gooding JJ, Gaus K (2011). Pre-existing clusters of the adaptor Lat do not participate in early T cell signaling events. Nat Immunol.

[CR116] Willig KI, Rizzoli SO, Westphal V, Jahn R, Hell SW (2006). STED microscopy reveals that synaptotagmin remains clustered after synaptic vesicle exocytosis. Nature.

[CR117] Wilmes S, Staufenbiel M, Lisse D, Richter CP, Beutel O, Busch KB, Hess ST, Piehler J (2012). Triple-color super-resolution imaging of live cells: resolving submicroscopic receptor organization in the plasma membrane. Angew Chem Int Ed Engl.

[CR118] Wolter S, Schüttpelz M, Tscherepanow M, van de Linde S, Heilemann M, Sauer M (2010). Real-time computation of subdiffraction-resolution fluorescence images. J Microsc.

[CR119] Wolter S, Löschberger A, Holm T, Aufmkolk S, Dabauvalle MC, van de Linde S, Sauer M (2012). rapidSTORM: accurate, fast open-source software for localization microscopy. Nat Methods.

[CR120] Wombacher R, Heidbreder M, van de Linde S, Sheetz MP, Heilemann M, Cornish VW, Sauer M (2010). Live-cell super-resolution imaging with trimethoprim conjugates. Nat Methods.

[CR121] Xu K, Babcock HP, Zhuang X (2012). Dual-objective STORM reveals three-dimensional filament organization in the actin cytoskeleton. Nat Methods.

[CR122] York AG, Ghitani A, Vaziri A, Davidson MW, Shroff H (2011). Confined activation and subdiffractive localization enables whole-cell PALM with genetically expressed probes. Nat Methods.

[CR123] Zessin PJ, Finan K, Heilemann M (2012). Super-resolution fluorescence imaging of chromosomal DNA. J Struct Biol.

[CR124] Zessin PJM, Krüger CL, Malkusch S, Endesfelder U, Heilemann M (2013). A hydrophilic gel matrix for single-molecule super-resolution microscopy. Opt Nanosc.

